# Supplementary material for: How does it all end? Trends and disparities in health at the end of life
Source: PLoS One. 2022 Jul 27;17(7):e0267551. doi: 10.1371/journal.pone.0267551 (PMC9328500; doi:10.1371/journal.pone.0267551)
Supplement: S1 Appendix — (DOCX) [file pone.0267551.s001.docx]

**Appendix Table 1.** Years out of last six years of life spent in each health state for decedents 65+, over time: Females. 95% confidence interval in parentheses. *Continued on next page.*

| **Year of interview** | **Self-rated health** | | | | |  | **Any IADL limitation** |  | **ADL limitations** | | | |
| --- | --- | --- | --- | --- | --- | --- | --- | --- | --- | --- | --- | --- |
|  | Excellent | Very good | Good | Fair | Poor |  |  |  | None | 1 | 2 | 3+ |
| **Females** |  | | | | | | | | | | | |
| 1987-1988 | 0.52 (0.47, 0.58) | 0.93 (0.86, 1.00) | 1.68 (1.60, 1.77) | 1.61 (1.53, 1.70) | 1.25 (1.17, 1.33) |  | -- |  | -- | -- | -- | -- |
| 1989-1990 | 0.58 (0.52, 0.63) | 0.98 (0.91, 1.06) | 1.78 (1.68, 1.87) | 1.53 (1.44, 1.62) | 1.14 (1.06, 1.22) |  | -- |  | -- | -- | -- | -- |
| 1991-1992 | 0.57 (0.51, 0.62) | 0.93 (0.86, 0.99) | 1.76 (1.67, 1.84) | 1.58 (1.50, 1.66) | 1.16 (1.09, 1.24) |  | -- |  | -- | -- | -- | -- |
| 1993-1994 | 0.49 (0.43, 0.54) | 0.92 (0.85, 0.99) | 1.89 (1.80, 1.99) | 1.54 (1.44, 1.63) | 1.16 (1.08, 1.24) |  | -- |  | -- | -- | -- | -- |
| 1995-1996 | 0.52 (0.45, 0.59) | 0.99 (0.90, 1.09) | 1.74 (1.62, 1.85) | 1.64 (1.52, 1.76) | 1.11 (1.02, 1.21) |  | -- |  | -- | -- | -- | -- |
| 1997-1998 | 0.38 (0.32, 0.44) | 0.99 (0.91, 1.07) | 1.91 (1.80, 2.01) | 1.67 (1.58, 1.78) | 1.05 (0.96, 1.14) |  | 2.21 (2.10, 2.32) |  | 4.90 (4.82, 4.99) | 0.30 (0.26, 0.35) | 0.21 (0.17, 0.25) | 0.59 (0.52, 0.66) |
| 1999-2000 | 0.38 (0.32, 0.43) | 1.00 (0.91, 1.09) | 1.87 (1.75, 1.98) | 1.65 (1.55, 1.76) | 1.11 (1.02, 1.20) |  | 2.16 (2.04, 2.27) |  | 4.80 (4.71, 4.90) | 0.27 (0.22, 0.32) | 0.26 (0.21, 0.31) | 0.67 (0.60, 0.74) |
| 2001-2002 | 0.40 (0.34, 0.46) | 0.99 (0.89, 1.07) | 1.94 (1.83, 2.06) | 1.67 (1.56, 1.79) | 1.00 (0.91, 1.09) |  | 2.18 (2.07, 2.30) |  | 4.89 (4.79, 4.98) | 0.30 (0.25, 0.35) | 0.21 (0.17, 0.26) | 0.60 (0.53, 0.67) |
| 2003-2004 | 0.39 (0.33, 0.46) | 0.98 (0.90, 1.08) | 2.03 (1.92, 2.15) | 1.58 (1.48, 1.69) | 1.01 (0.92, 1.10) |  | 2.10 (1.97, 2.21) |  | 4.84 (4.74, 4.94) | 0.31 (0.26, 0.37) | 0.23 (0.18, 0.28) | 0.62 (0.55, 0.69) |
| 2005-2006 | 0.40 (0.33, 0.47) | 1.06 (0.96, 1.16) | 1.93 (1.80, 2.05) | 1.71 (1.59, 1.84) | 0.90 (0.81, 1.01) |  | 2.05 (1.93, 2.19) |  | 4.92 (4.81, 5.02) | 0.26 (0.20, 0.32) | 0.18 (0.13, 0.23) | 0.65 (0.56, 0.74) |
| 2007-2008 | 0.34 (0.28, 0.41) | 0.98 (0.87, 1.09) | 2.00 (1.87, 2.13) | 1.60 (1.46, 1.73) | 1.08 (0.96, 1.20) |  | 2.24 (2.10, 2.39) |  | 4.73 (4.60, 4.84) | 0.29 (0.23, 0.36) | 0.30 (0.23, 0.36) | 0.69 (0.59, 0.78) |
|  |  |  |  |  |  |  |  |  |  |  |  |  |

**Appendix Table 1,** *continued*: Males*.*

| **Year of interview** | **Self-rated health** | | | | |  | **Any IADL limitation**^a^ |  | **ADL limitations**^a^ | | | |
| --- | --- | --- | --- | --- | --- | --- | --- | --- | --- | --- | --- | --- |
|  | Excellent | Very good | Good | Fair | Poor |  |  |  | None | 1 | 2 | 3+ |
| **Males** |  | | | | | | | | | | | |
| 1987-1988 | 0.55 (0.50, 0.61) | 0.90 (0.83, 0.96) | 1.79 (1.70, 1.87) | 1.55 (1.45, 1.63) | 1.22 (1.14, 1.29) |  | -- |  | -- | -- | -- | -- |
| 1989-1990 | 0.61 (0.54, 0.68) | 0.89 (0.82, 0.96) | 1.80 (1.71, 1.89) | 1.48 (1.40, 1.57) | 1.21 (1.13, 1.30) |  | -- |  | -- | -- | -- | -- |
| 1991-1992 | 0.55 (0.49, 0.61) | 0.95 (0.88, 1.02) | 1.71 (1.63, 1.80) | 1.49 (1.41, 1.57) | 1.30 (1.22, 1.38) |  | -- |  | -- | -- | -- | -- |
| 1993-1994 | 0.55 (0.50, 0.62) | 0.96 (0.88, 1.03) | 1.79 (1.70, 1.88) | 1.50 (1.41, 1.60) | 1.20 (1.12, 1.29) |  | -- |  | -- | -- | -- | -- |
| 1995-1996 | 0.49 (0.42, 0.56) | 0.97 (0.88, 1.07) | 1.83 (1.72, 1.95) | 1.58 (1.46, 1.69) | 1.13 (1.03, 1.23) |  | -- |  | -- | -- | -- | -- |
| 1997-1998 | 0.51 (0.45, 0.58) | 1.00 (0.92, 1.08) | 1.93 (1.83, 2.04) | 1.58 (1.47, 1.68) | 0.98 (0.89, 1.06) |  | 1.15 (1.06, 1.24) |  | 5.33 (5.25, 5.40) | 0.15 (0.11, 0.19) | 0.19 (0.15, 0.24) | 0.33 (0.28, 0.39) |
| 1999-2000 | 0.48 (0.42, 0.54) | 1.00 (0.92, 1.09) | 1.87 (1.76, 1.98) | 1.61 (1.50, 1.71) | 1.05 (0.96, 1.14) |  | 1.16 (1.06, 1.25) |  | 5.31 (5.24, 5.39) | 0.19 (0.14, 0.23) | 0.13 (0.09, 0.16) | 0.37 (0.31, 0.43) |
| 2001-2002 | 0.51 (0.43, 0.57) | 0.96 (0.88, 1.05) | 1.89 (1.78, 2.00) | 1.60 (1.49, 1.72) | 1.04 (0.96, 1.13) |  | 1.12 (1.03, 1.21) |  | 5.32 (5.24, 5.40) | 0.11 (0.08, 0.14) | 0.16 (0.12, 0.20) | 0.41 (0.35, 0.48) |
| 2003-2004 | 0.43 (0.37, 0.51) | 0.88 (0.78, 0.97) | 1.96 (1.84, 2.08) | 1.67 (1.56, 1.80) | 1.05 (0.96, 1.15) |  | 1.15 (1.06, 1.25) |  | 5.29 (5.20, 5.37) | 0.15 (0.11, 0.19) | 0.13 (0.09, 0.16) | 0.44 (0.38, 0.51) |
| 2005-2006 | 0.52 (0.44, 0.60) | 0.90 (0.80, 0.99) | 1.98 (1.84, 2.10) | 1.62 (1.50, 1.75) | 0.99 (0.89, 1.09) |  | 1.20 (1.10, 1.32) |  | 5.32 (5.23, 5.41) | 0.17 (0.12, 0.22) | 0.13 (0.09, 0.17) | 0.38 (0.31, 0.45) |
| 2007-2008 | 0.45 (0.37, 0.54) | 1.03 (0.92, 1.14) | 1.89 (1.76, 2.03) | 1.66 (1.52, 1.80) | 0.96 (0.85, 1.08) |  | 1.13 (1.01, 1.25) |  | 5.35 (5.25, 5.45) | 0.14 (0.09, 0.19) | 0.10 (0.07, 0.14) | 0.41 (0.33, 0.48) |
|  |  |  |  |  |  |  |  |  |  |  |  |  |

*Source*: Self-rated health from NHIS 1986-2014, IADL and ADL from NHIS 1997-2014 95% confidence intervals from 500 bootstrapped replications.

a. The NHIS began consistently asking respondents about ADL and IADL limitations in 1997.
