# Supplementary material for: How does it all end? Trends and disparities in health at the end of life
Source: PLoS One. 2022 Jul 27;17(7):e0267551. doi: 10.1371/journal.pone.0267551 (PMC9328500; doi:10.1371/journal.pone.0267551)
Supplement: S2 Appendix — (DOCX) [file pone.0267551.s002.docx]

**Appendix Table 2.** Years out of last six years of life spent in each health state for decedents 65+, by age at death, race, and educational attainment: Females.
95% confidence interval in parentheses. *Continued on next page.*

| **Characteristic** | **Self-rated health** | | | | |  | **Any IADL limitation** |  | **ADL limitations** | | | |
| --- | --- | --- | --- | --- | --- | --- | --- | --- | --- | --- | --- | --- |
|  | Excellent | Very good | Good | Fair | Poor |  |  |  | None | 1 | 2 | 3+ |
| **Females** |  | | | | | | | | | | | |
| **Overall** | 0.39  (0.37, 0.42) | 1.03  (0.99, 1.06) | 1.96  (1.92, 2.00) | 1.65  (1.61, 1.69) | 0.98  (0.95, 1.01) |  | 2.17  (2.13, 2.21) |  | 4.82  (4.78, 4.85) | 0.28  (0.26, 0.30) | 0.23  (0.22, 0.25) | 0.67  (0.64, 0.70) |
|  |  |  |  |  |  |  |  |  |  |  |  |  |
| **Age at death** |  |  |  |  |  |  |  |  |  |  |  |  |
| 65-74 | 0.42  (0.37, 0.46) | 0.91  (0.84, 0.98) | 1.79  (1.70, 1.87) | 1.70  (1.62, 1.79) | 1.18  (1.10, 1.26) |  | 1.47  (1.38, 1.55) |  | 5.22  (5.15, 5.28) | 0.16  (0.12, 0.19) | 0.17  (0.14, 0.20) | 0.46  (0.40, 0.52) |
| 75-84 | 0.35  (0.31, 0.38) | 1.02  (0.96, 1.08) | 1.93  (1.86, 2.01) | 1.69  (1.62, 1.76) | 1.01  (0.96, 1.07) |  | 1.85  (1.78, 1.92) |  | 4.98  (4.92, 5.04) | 0.24  (0.21, 0.27) | 0.21  (0.19, 0.24) | 0.56  (0.52, 0.61) |
| 85+ | 0.42  (0.39, 0.45) | 1.09  (1.04, 1.15) | 2.07  (2.01, 2.15) | 1.58  (1.51, 1.64) | 0.84  (0.79, 0.89) |  | 2.82  (2.76, 2.89) |  | 4.45  (4.38, 4.51) | 0.38  (0.35, 0.42) | 0.29  (0.26, 0.32) | 0.88  (0.83, 0.93) |
|  |  |  |  |  |  |  |  |  |  |  |  |  |
| **Race** |  |  |  |  |  |  |  |  |  |  |  |  |
| Non-Hisp. white | 0.41  (0.39, 0.44) | 1.11  (1.07, 1.15) | 2.01  (1.96, 2.06) | 1.56  (1.51, 1.60) | 0.91  (0.87, 0.95) |  | 2.12  (2.07, 2.17) |  | 4.90  (4.86, 4.93) | 0.29  (0.26, 0.31) | 0.22  (0.20, 0.24) | 0.59  (0.57, 0.62) |
| Non-Hisp. black | 0.27  (0.21, 0.32) | 0.66  (0.59, 0.73) | 1.63  (1.53, 1.74) | 2.12  (1.99, 2.23) | 1.32  (1.21, 1.42) |  | 2.52  (2.40, 2.63) |  | 4.40  (4.30, 4.49) | 0.28  (0.24, 0.34) | 0.29  (0.24, 0.35) | 1.03  (0.93, 1.12) |
|  | |  |  |  |  |  |  |  |  |  |  |  |
| **Educational attainment** | |  |  |  |  |  |  |  |  |  |  |  |
| <High school | 0.27  (0.23, 0.30) | 0.77  (0.72, 0.82) | 1.79  (1.73, 1.86) | 1.90  (1.83, 1.97) | 1.28  (1.21, 1.35) |  | 2.47  (2.40, 2.55) |  | 4.67  (4.61, 4.73) | 0.32  (0.28, 0.35) | 0.26  (0.23, 0.30) | 0.76  (0.70, 0.80) |
| HS/Some coll. | 0.42  (0.39, 0.46) | 1.13  (1.08, 1.18) | 2.05  (1.98, 2.11) | 1.57  (1.52, 1.63) | 0.83  (0.79, 0.88) |  | 2.02  (1.97, 2.07) |  | 4.94  (4.90, 4.99) | 0.25  (0.23, 0.28) | 0.21  (0.19, 0.24) | 0.59  (0.55, 0.63) |
| BA or more | 0.65  (0.56, 0.75) | 1.41  (1.29, 1.54) | 2.07  (1.93, 2.21) | 1.21  (1.11, 1.34) | 0.66  (0.56, 0.75) |  | 1.83  (1.70, 1.98) |  | 4.95  (4.85, 5.06) | 0.23  (0.18, 0.29) | 0.20  (0.15, 0.25) | 0.62  (0.54, 0.70) |
|  |  |  |  |  |  |  |  |  |  |  |  |  |

**Table 2,** *continued*: Males*.*

| **Characteristic** | **Self-rated health** | | | | |  | | **Any IADL limitation** | |  | | **ADL limitations** | | | | |  |
| --- | --- | --- | --- | --- | --- | --- | --- | --- | --- | --- | --- | --- | --- | --- | --- | --- | --- |
|  | Excellent | Very good | Good | Fair | Poor | |  | |  | |  | | None | 1 | 2 | 3+ | |
| **Males** |  | | | | | | | | | | | | | | | |  |
| **Overall** | 0.48  (0.46, 0.51) | 0.97  (0.94, 1.01) | 1.93  (1.89, 1.97) | 1.63  (1.59, 1.68) | 0.98  (0.95, 1.01) | |  | | 1.16  (1.13, 1.19) | |  | | 5.31  (5.28, 5.33) | 0.15  (0.14, 0.17) | 0.14  (0.13, 0.15) | 0.40  (0.38, 0.42) | |
|  |  |  |  |  |  | |  | |  | |  | |  |  |  |  | |
| **Age at death** |  |  |  |  |  | |  | |  | |  | |  |  |  |  | |
| 65-74 | 0.54  (0.49, 0.59) | 0.91  (0.86, 0.98) | 1.77  (1.69, 1.84) | 1.63  (1.56, 1.72) | 1.15  (1.08, 1.21) | |  | | 0.85  (0.79, 0.91) | |  | | 5.48  (5.44, 5.53) | 0.13  (0.10, 0.15) | 0.10  (0.08, 0.12) | 0.29  (0.26, 0.33) | |
| 74-84 | 0.43  (0.40, 0.48) | 0.96  (0.91, 1.02) | 1.94  (1.87, 2.01) | 1.67  (1.61, 1.74) | 1.00  (0.94, 1.05) | |  | | 1.03  (0.98, 1.08) | |  | | 5.35  (5.30, 5.39) | 0.13  (0.11, 0.16) | 0.13  (0.11, 0.15) | 0.38  (0.35, 0.42) | |
| 85+ | 0.48  (0.43, 0.53) | 1.05  (1.00, 1.12) | 2.09  (2.01, 2.17) | 1.59  (1.52, 1.66) | 0.79  (0.73, 0.84) | |  | | 1.66  (1.58, 1.73) | |  | | 5.07  (5.00, 5.13) | 0.21  (0.18, 0.24) | 0.19  (0.16, 0.22) | 0.54  (0.49, 0.59) | |
|  |  |  |  |  |  | |  | |  | |  | |  |  |  |  | |
| **Race** |  |  |  |  |  | |  | |  | |  | |  |  |  |  | |
| Non-Hisp. white | 0.50  (0.47, 0.53) | 1.02  (0.98, 1.06) | 1.96  (1.91, 2.01) | 1.58  (1.54, 1.63) | 0.94  (0.91, 0.98) | |  | | 1.13  (1.09, 1.17) | |  | | 5.34  (5.31, 5.37) | 0.16  (0.14, 0.18) | 0.14  (0.12, 0.15) | 0.36  (0.34, 0.39) | |
| Non-Hisp black | 0.30  (0.23, 0.35) | 0.71  (0.63, 0.80) | 1.85  (1.72, 1.97) | 1.98  (1.85, 2.11) | 1.17  (1.07, 1.27) | |  | | 1.40  (1.30, 1.51) | |  | | 5.21  (5.12, 5.30) | 0.13  (0.09, 0.17) | 0.16  (0.12, 0.20) | 0.50  (0.42, 0.57) | |
|  | |  |  |  |  | |  | |  | |  | |  |  |  |  | |
| **Educational attainment** | |  |  |  |  | |  | |  | |  | |  |  |  |  | |
| <High school | 0.32  (0.28, 0.35) | 0.70  (0.65, 0.76) | 1.75  (1.68, 1.82) | 1.94  (1.87, 2.02) | 1.29  (1.22, 1.36) | |  | | 1.37  (1.31, 1.44) | |  | | 5.27  (5.23, 5.32) | 0.16  (0.13, 0.18) | 0.14  (0.12, 0.17) | 0.43  (0.38, 0.47) | |
| HS/Some coll. | 0.48  (0.44, 0.51) | 1.02  (0.97, 1.07) | 2.03  (1.96, 2.10) | 1.58  (1.53, 1.64) | 0.89  (0.85, 0.94) | |  | | 1.04  (1.00, 1.09) | |  | | 5.34  (5.30, 5.38) | 0.14  (0.12, 0.16) | 0.14  (0.12, 0.16) | 0.38  (0.35, 0.41) | |
| BA or more | 0.78  (0.70, 0.86) | 1.34  (1.25, 1.44) | 1.98  (1.88, 2.10) | 1.25  (1.16, 1.35) | 0.65  (0.58, 0.71) | |  | | 1.04  (0.96, 1.13) | |  | | 5.35  (5.27, 5.42) | 0.18  (0.14, 0.23) | 0.10  (0.07, 0.12) | 0.37  (0.31, 0.43) | |

*Source*: Self-rated health from NHIS 1986-2014, IADL and ADL from NHIS 1997-2014 95% confidence intervals from 500 bootstrapped replications.
