# Supplementary material for: How does it all end? Trends and disparities in health at the end of life
Source: PLoS One. 2022 Jul 27;17(7):e0267551. doi: 10.1371/journal.pone.0267551 (PMC9328500; doi:10.1371/journal.pone.0267551)
Supplement: Attachment — Submitted filename: Manuscript PONE-D-21-16309.docx [file pone.0267551.s003.docx]

**Manuscript: PONE-D-21-16309**

**How does it all end? Trends and disparities in health at the end of life**

Thank you for the opportunity to review this research article that considers trends and disparities in quality of life prior to death among adults age 65 and older in the U.S. The author investigates self-rated health (SRH) and disability in the last six years of life across: 1) time, 2) population subgroups (i.e., Black-White and male-female), and 3) causes of death. The author uses data from the 1987-2014 National Health Interview Survey, linked to death records through 2015, for respondents who died within six years of being interviewed. Decedents are classified as having a disability prior to death based on needing help with at least one of six ADLs and/or needing help with routine needs, such as grocery shopping or doing light chores. Causes of death are classified as: 1) accidents, 2) cancers, 3) cerebrovascular diseases, 4) chronic lower respiratory diseases, 5) a combination of heart disease and diabetes, and 6) all other causes. The manuscript is fairly well written and addresses an important topic (i.e., trends and disparities in health at end of life). Findings from such a study have the potential to inform interventions and guide program planning and address disparities. The author indicates that this study is the first known study to investigate trends in SRH in the last years of life which can make an important contribution to the literature. Some concerns noted below limit the potential impact of the paper.

1. The author should be more clear regarding data that are used. The abstract and Findings refer to data from the 1987-2014 National Health Interview Survey (NHIS) whereas the Methods (p. 5) refer to data from the 1997-2014 NHIS.
2. The rationale for using a timeframe of up to six years-to-death is unclear. How was the six years-to-death timeframe determined?
3. Given the focus on disparities among subgroups, including investigating Black-White differences, the author might consider an earlier paper by Liao et al., (1999) that also used NHIS data and can provide important context for the current paper. Using data from the 1986-1994 NHIS, linked to death records through 1995, these researchers investigated Black-White differences in disability and mortality in decedents age 50 years and older who died within 2 years of their interview. Researchers found that adjusting for educational attainment (used as a proxy for socioeconomic status) did not eliminate Black-White differences in disability and morbidity in the last years of life but did account for much of the difference. The current paper considers differences in health status with regard to race as well as education and gender but does not adjust for any potential confounding by socioeconomic status or other social determinants with regard to Black-White differences which might be important in informing interventions to address modifiable factors associated with these determinants.
4. A seminal paper by Lunney et al. (2003) that also is not cited by the authors investigated differences in functional decline among four types of illness trajectories: 1) sudden death, 2) cancer death, 3) death from organ failure, and 4) frailty. This research found that patterns of functional decline or disability varied substantially based on illness trajectory. Similar to the current paper, decedents who died suddenly or who died from cancer experienced the shortest periods of disability prior to death. Given that variations in the shape of disability trajectories by disease type is well established, findings related to cause of death in the current paper are not surprising or particularly compelling. Examining differences related to number of chronic conditions and their disabling effects at end of life might be more informative, especially since some evidence indicates that these disabling effects (i.e., based on number of chronic conditions) seem to be similar for Blacks and Whites who are approaching death.
5. Related to # 4 above, a large percentage of the sample (43% of females and 51% of males) died from accidents and cancers (shown in previous studies to be associated with the shortest periods of disability prior to death) and 41% of females and 35% of males are classified as “all other” causes of death. Only about 16% of females and 14% of males are grouped into specific non-cancer disease categories. It also is unclear why decedents with heart disease and diabetes are grouped into one category, even if people with diabetes are at high risk for heart disease. These cause of death categories do not seem particularly meaningful or informative.
6. Related to #4 and #5 above, the author does not indicate how overlaps in chronic conditions are handled. Given that multiple chronic conditions are common among people age 65 and older, it is likely that many decedents reported more than one chronic condition.
7. Related to #4, #5, and #6 above, the Liao et al., (1999) paper (referenced above under #3) that also used NHIS data to investigate disparities and trends in disability in the last years of life excluded decedents who died from accidental causes. The author might want to also consider excluding these decedents. They do not seem theoretically relevant. It also would be useful for the author to better describe how information about chronic conditions is collected in the NHIS. What are the range and types of conditions reflected in the survey?
8. On p. 15, the authors indicates not including Alzheimer’s disease as a cause of death in the analysis based on the high likelihood of institutionalization with disease progression. However, the author indicates on p. 15 that decedents who enter a care facility after their interview are retained in the sample. Again, the rationale for the cause of death categories used in this study is questionable.
9. In addition, it would be useful to have more information regarding the measure of IADLs used in this study beyond just grocery shopping or doing light chores. Help with grocery shopping can be related to a lack of transportation as opposed to disability. The six ADLs are listed but less information is provided about the IADL measure.
