# Supplementary material for: How does it all end? Trends and disparities in health at the end of life
Source: PLoS One. 2022 Jul 27;17(7):e0267551. doi: 10.1371/journal.pone.0267551 (PMC9328500; doi:10.1371/journal.pone.0267551)
Supplement: Attachment — Submitted filename: Response to Reviewers.docx [file pone.0267551.s005.docx]

**Response to Reviewers**

Dear Professor Idler and Reviewers,

I would like to thank you again for the time you have taken to provide constructive feedback. As before, I below respond to each point with AR (for “Author’s Response”).

Best,

The Author

**Reviewer #1**

Thank you for the opportunity to review this revised manuscript that considers trends and disparities in end-of-life health in the U.S. The author made a good effort to address both reviewers’ (and the editor’s) comments/concerns and has strengthened the paper. Below, I have outlined some additional points to consider. Some additional editing and wordsmithing are needed and would further improve the quality of the paper.

1. The abstract could use some editing. For example, the sentence: “Time spent in fair/poor health over years 1987-2008 declined by two months, while time lived with at least one activity limitation generally remained stable from 1997-2008” is a little clunky. Time spent in fair/poor health over years 1987-2008 declined by two months compared to what? Declined by two months each year? The sentences that follow also could use some editing. The sentence: “Compared to men, women reported an IADL for 1 year longer and an ADL for 8 extra months, yet both sexes reported similar lengths of unfavorable health” also needs editing. The numbers 1 and 8 should be spelled out. Reported an IADL or ADL what? Limitation? what does the author mean by “similar lengths of unfavorable health? The way that the sentence is currently written, it is not clear what is meant here. In the next sentence, I suggest saying similar health “compared with” younger decedents instead of “to” younger decedents. Given the use of repeated cross-sectional data, I suggest using language such as “findings indicate” rather than stating the findings as if they are fact in the Discussion section of the Abstract.

AR: Thank you for these suggestions. I have significantly re-written the Results and Discussion sections of the abstract.

2. On page 4, the author indicates the importance of examining ADL limitations (e.g., 40% of people over age 65 have at least one limitation and nearly 90% with 3+ limitations require caregiving help) but do not say why it might be important to also consider IADL limitations. They do make the case on p. 10 and may want to say something similar here on p. 4 as well. The sentence: “ADL limitations are predictive of requiring physical assistance, with roughly 40% of community-dwelling adults age 65+ with one limitation and nearly 90% with 3+ receiving caregiving help” needs editing to read more clearly. Also, physical assistance is caregiving help.

AR: In addition to clarifying the suggested sentence, I have added a sentence to expand on the utility of IADL limitations:

“While IADL limitations are less disabling than ADL limitations, an IADL limitation indicates that an individual requires some level of support in order to live independently.”

3. On p. 5, what is meant by the sentence: “Because of these simultaneously evolving environments and population characteristics, it is difficult to separate period and cohort effects.” I am not following this line of thought. I agree that the methods used in this paper (and acknowledged under Limitations) limit the ability to disentangle these effects.

AR: I have re-written the sentence to read:

“Because cohorts are evolving at the same time as the contexts in which they live, it is difficult to separate period and cohort effects.”

4. On p. 7, instead of using wording such as, “an older study similarly constructed to this one,” consider, for example, referring to “a similar study” or “another study with a similar design,” etc. The wording here is clunky.

AR: I have made the change to “a similar study”.

5. In describing the sample on p. 9 in the Methods section, information should be included regarding any exclusion criteria (e.g., living in a long-term care facility at baseline). Information should also be included here regarding the possibility that those not in long-term care at baseline could be included at follow up.

AR: I have added the following sentence:

“While residents of long-term care institutions are not included in the baseline sample, the sample may include individuals who were interviewed at home and then moved to an institution during the follow-up period.”

6. The literature reported in the first paragraph under the section on "Years-to death” on p. 9 in the Methods section feels out of place here. This literature should be woven into the Background section that begins on p. 3 and not here. A Methods section typically does not include a literature review. The rationale for the six years-to death timeframe can be made without including all of these citations in the Methods section.

AR: As you suggest, I have moved the section with the citations to the Background section, while maintaining a shortened rationale for the time frame in the Methods section.

7. On p. 12, I suggest more clearly outlining how data were pooled to conduct the varying analyses and describing the different analytic approaches in turn rather than stating: “To compare estimates across population subgroups, I pool data across years 1997-2014 (1987-2014 for SRH).”

AR: I have rewritten this sentence and avoided using the term “pooling”, which made the approach sound unnecessarily complicated. The sentence now reads:

“To compare outcomes across population subgroups, I estimate the mean of an outcome prevalence across the study period (years 1997-2014 for SRH; years 1997-2014 for IADL and ADL).”

8. Avoid using language like, “women reported either kinds of limitations for much longer” on p. 14. Again, the paper could benefit from some wordsmithing throughout to improve readability and flow and to sound more academic.

AR: I have adjusted the wording and gone through the manuscript to find a balance between accessibility and sounding academic.

9. On p. 21, avoid using the term “elder.” The term, “older adults” is preferred by aging researchers and others.

AR: I have the term changed “elder care” to “care for older adults.”

10. When referring to Blacks and Whites, I suggest capitalizing both terms to be consistent.

AR: Thank you for this suggestion. I gave careful thought to my decision to capitalize Black, but not white, in my past submissions of the manuscript. The stylistic debate today is of course tied to discussions around identity and racism in the US today.

Around the time of my first submission in 2021, the [Associated Press announced](https://blog.ap.org/announcements/why-we-will-lowercase-white) that it would capitalize Black, and not white.

This thought piece in [*The Atlantic*](https://www.theatlantic.com/ideas/archive/2020/06/time-to-capitalize-blackand-white/613159/) also gives a good overview of the current debate.

On reconsideration of the current debate (and in particular [this piece](https://zora.medium.com/im-a-black-scholar-who-studies-race-here-s-why-i-capitalize-white-f94883aa2dd3) by Dr. Eve Ewing), I have chosen to capitalize White.

The initial aim was of the typographical inconsistency was to highlight the upper case B. A prominent argument against the lowercase w, however, is that its use doesn’t recognize Whiteness as a sociological construct and allows White people to sit out on the debates and work around racism.

Therefore, I have made the change throughout.

11. Throughout the body of the text, “vs.” should be written out as “versus” unless included in material with parentheses.

AR: Thank you. I have made two necessary changes.

12. On p. 11, instead of “non-Black and non-white racial/ethnic categorizations,” I might indicate that sample sizes for other racial and ethnic groups were too small to conduct any additional racial/ethnic comparisons. The language used here is a little clunky.

AR: Good point. The sentence now reads:

“Due to the small sample sizes for other racial/ethnic categorizations, …”

13. On pp. 8-11, text indicates that different sample sizes were used for different analyses (e.g., SRH vs. ADLs and IADLs as well as racial comparisons vs. other types of comparisons (e.g., educational attainment). It is not clear what the different sample sizes were for the various analyses.

AR: Thank you for pointing this out. The information was previously buried in the notes for Table 1. I now mention this in a footnote in the section describing the sample size:

“The SRH analysis sample consists of 77,295 individuals across all years 1987-2014. Sample sizes for the IADL and ADL analyses, which span years 1997-2014, are 40,354 and 40,359, respectively.”

14. On p. 23, The author indicates that “measuring the relative importance of cohort composition vs. period changes in the treatment of illness,” etc. would help target relevant interventions.” A brief example of how this type of analysis could inform relevant interventions might be useful here. As noted by Reviewer 2, the methods used and inability to disentangle age versus cohort versus period effects is a limitation that should be acknowledged. The author acknowledges this limitation but does not provide a concrete explanation of how/why the analysis is limited because of this constraint.

AR: Thank you for the suggestion. I have added the second sentence:

“Measuring the relative importance of cohort composition versus period changes in the treatment of illness and disability would help target relevant interventions. For example, knowing that older male cohorts are less likely than their predecessors to be heavy smokers (Preston & Wang 2006), but more likely to face the health problems associated with obesity (Wang et al 2011), could inform the decision to divert funds away from tobacco-related interventions and toward new programs targeting obesity.”

15. In the conclusion on p. 23, I suggest saying something like, “findings indicate”, etc., rather than stating results as fact.

AR: I now use weaker language throughout the discussion.

Reviewer #2: The authors have done a very thorough and responsive revision. I have just a few remaining suggestions for improvement.

1. The manuscript would benefit from a very careful copy-edit both for clarity and style. For instance, the paper opens on an awkward note. The first sentence of a manuscript should be more direct, such as “Death—and the months, days, or moments preceding it—are an important and distinct stage of the life course (Cohen-Mansfield et al. 2018)” (deleting initial clause).

AR: Thank you for the suggestion. I have adjusted the first sentence and have made clarifying changes throughout the manuscript.

2. A recent paper by Carr and Luth (2019) Annual Review of Sociology makes the case that ‘end of life’ is a distinctive life course stage. This article could be helpful for your framing of the analysis.

AR: I now cite this helpful paper throughout the manuscript.

3. The front end of the paper could do a bit more to motivate the selection of the multiple health measures. These issues are addressed somewhat in the Discussion, but it would be helpful to foreground the fact that SRH is subjective and assessed in relation to one’s peers (who may be dying or in poor health), whereas the IADL and ADL measures are more ‘objective’ and reflect behavioral capacities.

AR: Thank you for this suggestion. The Background section now reads:

“SRH is a subjective and self-reported indicator of health. While disability is also self-reported, it serves as a more objective measure of requiring assistance.”

Overall, an interesting and creative paper that will make a nice contribution to the literature.a
